# Machine learning-based endoplasmic reticulum-related diagnostic biomarker and immune microenvironment landscape for osteoarthritis

**DOI:** 10.18632/aging.205611

**Published:** 2024-02-28

**Authors:** Tingting Liu, Xiaomao Li, Mu Pang, Lifen Wang, Ye Li, Xizhe Sun

**Affiliations:** 1 Research Center for Drug Safety Evaluation of Hainan, Hainan Medical University, Haikou, Hainan 571199, China; 2 Jiangsu Food and Pharmaceutical Science College, Huaian, Jiangsu 223023, China; 3 The Fourth Clinical Medical College of Guangzhou University of Chinese Medicine (Shenzhen Traditional Chinese Medicine Hospital), Shenzhen, Guangdong 518000, China; 4 Chongqing Three Gorges Medical College, Chongqing 404120, China

**Keywords:** osteoarthritis, endoplasmic reticulum, diagnostic biomarker, immune microenvironment, machine learning

## Abstract

Background: Osteoarthritis (OA) is the most common degenerative joint disease worldwide. Further improving the current limited understanding of osteoarthritis has positive clinical value.

Methods: OA samples were collected from GEO database and endoplasmic reticulum related genes (ERRGs) were identified. The WGCNA network was further built to identify the crucial gene module. Based on the expression profiles of characteristic ERRGs, LASSO algorithm was used to select key factors according to the minimum λ value. Random forest (RF) algorithm was used to calculate the importance of ERRGs. Subsequently, overlapping genes based on LASSO and RF algorithms were identified as ERRGs-related diagnostic biomarkers. In addition, OA specimens were also collected and performed qRT-PCR quantitative analysis of selected ERRGs.

Results: We identified four ERRGs associated with OA risk assessment through machine learning methods, and verified the abnormal expressions of these screened markers in OA patients through *in vitro* experiments. The influence of selected markers on OA immune infiltration was also evaluated.

Conclusions: Our results provide new evidence for the role of ER stress in the OA progression, as well as new markers and potential intervention targets for OA.

## INTRODUCTION

Osteoarthritis (OA) is the most common degenerative joint disease worldwide, characterized by symptoms such as cartilage degradation, pain and limited movement, accompanied by a significant decline in the patient’s quality of life [1]. There are currently no pharmacological methods to inhibit disease progression or reduce cartilage damage. OA treatment strategies are limited to adjunctive therapy such as joint replacement surgery and physical therapy to improve function [2]. Further improving the current limited understanding of OA has positive clinical value.

Due to the heterogeneity of OA, individual etiology, clinical manifestations and response to treatment were inconsistent [3, 4]. These present great challenges for the prevention, diagnosis and prognosis prediction of OA. As objective, quantifiable features, biomarkers contribute to disease risk assessment by analyzing measurement reliability and summarizing biological, physiological or pathological pathways [5]. Currently, the only clinically used OA biomarkers are imaging markers. However, imaging markers cannot detect molecular alterations prior to the appearance of structural changes or provide potential therapeutic targets [6]. The predictive reliability of currently discovered OA biomarkers related to cartilage/bone structure, inflammation, and metabolism also requires further careful consideration [7–9]. At the same time, the reliability of multiple marker combinations in predicting OA severity and progression was significantly higher than that of a single marker [10, 11]. Therefore, there is an urgent need to screen for new meaningful diagnostic and prognostic markers.

Endoplasmic reticulum (ER) stress is caused by impaired protein folding capacity in ER, leading to accumulation of incorrectly folded proteins in ER, which adversely affects cell physiological function [12]. There is a lot of evidence showing the role of ER stress in the development of OA. Chondrodysplasia is usually caused by mutations in genes that code for cartilage components. This genetic mutation causes impaired synthesis or secretion of extracellular matrix (ECM) components that make up the main part of cartilage and aggregate in ER, ultimately leading to loss of ECM and disruption of chondrocyte homeostasis [13–15]. As two major risk factors for OA, aging and obesity both impair the function of key ER molecular chaperones, leading to improper protein folding and ER stress [16]. Thus, ER stress-induced chondrocyte death has been identified as a contributing factor to OA and a potentially reliable treatment strategy [17]. It is necessary to further explore the mechanism and clinical value of ER stress in OA.

The correlation between endoplasmic reticulum stress and immune infiltration has also been gradually clarified. Further activation of the unfolded protein response (UPR) during endoplasmic reticulum stress is characteristic of many autoimmune diseases [18]. Microenvironmental stress in the immune-infiltrating microenvironment, including hypoxia, reactive oxygen species, and pro-inflammatory cytokines, may increase the level of endoplasmic reticulum stress in dendritic cells (DC) and fibroblast-like synovial cells (FLS) in joints [19]. There is evidence that endoplasmic reticulum stress plays a role in the development process from B cells to plasma cells and the secretion of immunoglobulin [20, 21]. In addition, accumulation of misfolded proteins during ER stress can lead to increased MHC presentation on the cell surface, thereby increasing the chance of auto-reactive T cell activation [18]. Several pro-inflammatory cytokines have also been reported to act through endoplasmic reticulum stress processes [22, 23]. Therefore, the influence of endoplasmic reticulum stress on the immune infiltration of adaptive immune cells and related genes are worthy of further exploration.

In this study, we identified four endoplasmic reticulum related genes (ERRGs) associated with OA risk assessment through machine learning methods, and verified the abnormal expression of these screened markers in OA patients through *in vitro* experiments. At the same time, we evaluated the effect of markers on OA immune infiltration. Our results provide new evidence for the role of ER stress in the progression of OA, as well as new markers and potential intervention targets for OA.

## MATERIALS AND METHODS

### Data collection and pre-processing

Three open access datasets of OA and HC samples were collected from the GEO database, including GSE51588, GSE98918 and GSE117999. The “limma” script was used to preprocess the raw data of each GEO dataset and “SVA” script was utilized to normalize the raw data and eliminate the batch effect of the three GEO datasets in the R language environment [24]. In this study, the endoplasmic reticulum related genes (ERRGs) were identified based on the GeneCards database [25] (Supplementary Table 1).

### Differential expression analysis of transcriptome data and molecular pathways enrichment prediction

After the normalization of the transcriptome data of HC and OA samples, we conducted a differential expression analysis via “limma” script in the R environment. Under the selection criteria of *p*.adjust < 0.05, the DEGs between the OA and HC groups was identified. Based on the SRTING database, the potential interaction of ERRGs was revealed using the “string” script. Moreover, we used the “clusterProfiler” package to predict the potential molecular function and KEGG pathways of the ERRGs.

### Construction of WGCNA network to identify the crucial gene module for OA

A WGCNA network model was established to identify the crucial gene module for OA. In first, according to the transcriptome data of all samples (HC samples and OA samples), we developed a clustering tree to exclude the abnormal samples. Next, based on the optimal soft threshold (β), we established a WGCNA network. After the exclusion filter of expression level was set at 0.5, the genes were divided into the different gene modules and the dynamic tree cut method to merge the similar gene modules. Pearson correlation was used to evaluate the relationship between each gene module and estimate the potential correlation between clinical trait and each gene module. Finally, the most relevant gene module was chosen for the subsequent analysis.

### Generation of ERRG related diagnostic biomarker based on the machine learning algorithm

Two unique machine learning algorithms were utilized to identify the ERRGs related diagnostic biomarkers. Based on the expression profiler of the feature ERRGs related genes, a LASSO algorithm was performed to select the pivotal ERRG related genes according to the minimum λ value. Random forest (RF) algorithm was conducted to calculate the importance of each feature ERRGs related genes. Subsequently, the overlapping genes based on LASSO and RF algorithms were identified as the ERRGs related diagnostic biomarker.

### Model diagnostic effectiveness evaluation of ERRGs related biomarker and nomogram

R package “rms” was used to establish the nomogram model based on the expression profiler of ERRGs related biomarkers. The formula for nomogram was: nomogram score = HSPA5 × −5.4 + UBL4A × 5.2 + ATF4 × −4.9 + PPP1R15A × −4.4. Utilizing the “pROC” script, the ROC curve was carried out to evaluate the model diagnostic effectiveness of the ERRGs related biomarkers for OA.

### Characterization of immune microenvironment landscape

According to the 22 immune cell gene markers signature, we used the CIBERSORT estimation algorithm to evaluate the immune cell relative percent of OA and HC samples. In addition, Pearson correlation method was performed to estimate the potential correlation of ERRGs related biomarkers and 22 immune cells.

### qRT-PCR quantitative analysis of ERRGs related biomarkers

Real-time fluorescence quantitative qPCR was performed to detect the expression levels of HSPA5, UBL4A, ATF4, and PPP1R15A. Frozen OA tissues were taken out at −80°C, and total RNA was extracted, and all the steps were strictly in accordance with the instructions of the RNA Extraction Kit (Qiagen, Germany). The RNA concentration of each sample was determined by Nanodrop 2000, and RNA was reverse transcribed into cDNA according to the Reverse Transcription Kit (TaKaRa, Japan). cDNA was then used as a template for amplification of HSPA5, UBL4A, ATF4 and PPP1R15A on a Bio-Rad CFX90 Real-time PCR instrument. qRT-PCR reaction conditions were as follows: 95°C pre-denaturation for 30 s; 95°C denaturation for 5 s, RT-PCR reaction conditions were as follows: 95°C pre-denaturation for 30 s; 95°C denaturation for 5 s, and 95°C denaturation for 5 s. The reaction was performed at the same time as the reaction. The reaction conditions were: pre-denaturation at 95°C for 30 s; denaturation at 95°C for 5 s; annealing at 56°C for 5 s; and extension at 65°C for 5 s. A total of 39 cycles were performed. The relative expression of target genes was analyzed by using 2^−ΔΔCt^ (ΔCt = Ct value of target gene - Ct value of internal reference), and GAPDH was used as internal reference (Supplementary Table 2).

### Statistical analysis

All statistical analysis were performed under the R software 4.1.0 (https://cran.r-project.org/) and Perl language environment. Statistical comparison of data between the two groups was calculated using “limma” R package (Wilcox rank-sum test). *p* < 0.05 was considered to be statistically significant.

### Data availability statement

The bioinformatic datasets presented in this study can be found in online repositories. The names of the online repositories and accession numbers can be found in the article.

## RESULTS

### Data processing and differential expression analysis of OA and HC groups

**Figure 1:**
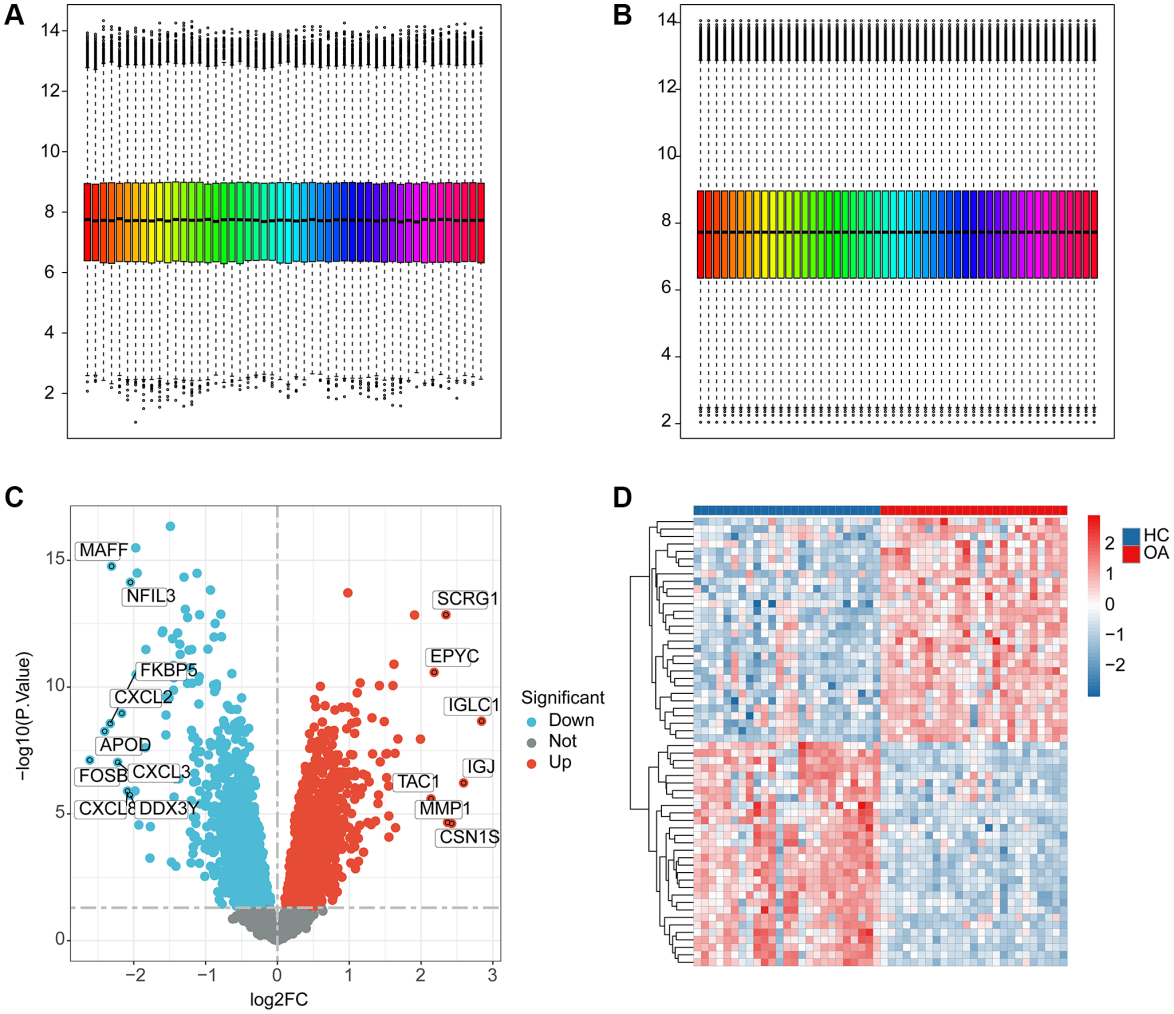
**The workflows of data processing and differential expression analysis between HC and OA groups.** (**A**, **B**) Data pre-processing of HC and OA samples in GEO database. (**C**) Identification of the DEGs between the HC and OA samples, the red dot indicates the up-DEGs and the blue dots indicate the down-DEGs in OA. The standard for selecting DEGs is set at *p*.adjust < 0.05. (**D**) The expression analysis of DEGs between HC and OA groups.

We extracted a total of 25 HC samples and 25 OA samples from the GEO database to investigate the potential function of ERAGs in OA. Utilizing the “sva” software, the batch effect of each sample was removed and normalized (Figure 1A, 1B). According to the “limma” package, the differential expression analysis was performed with the analysis standards set at *p*.adjust < 0.05 and |FC|>1 (Figure 1C). The heatmap reveals the most significantly DEGs between the OA and HC groups (Figure 1D).

### Identification of the pivotal gene module associated with OA via WGCNA

**Figure 2:**
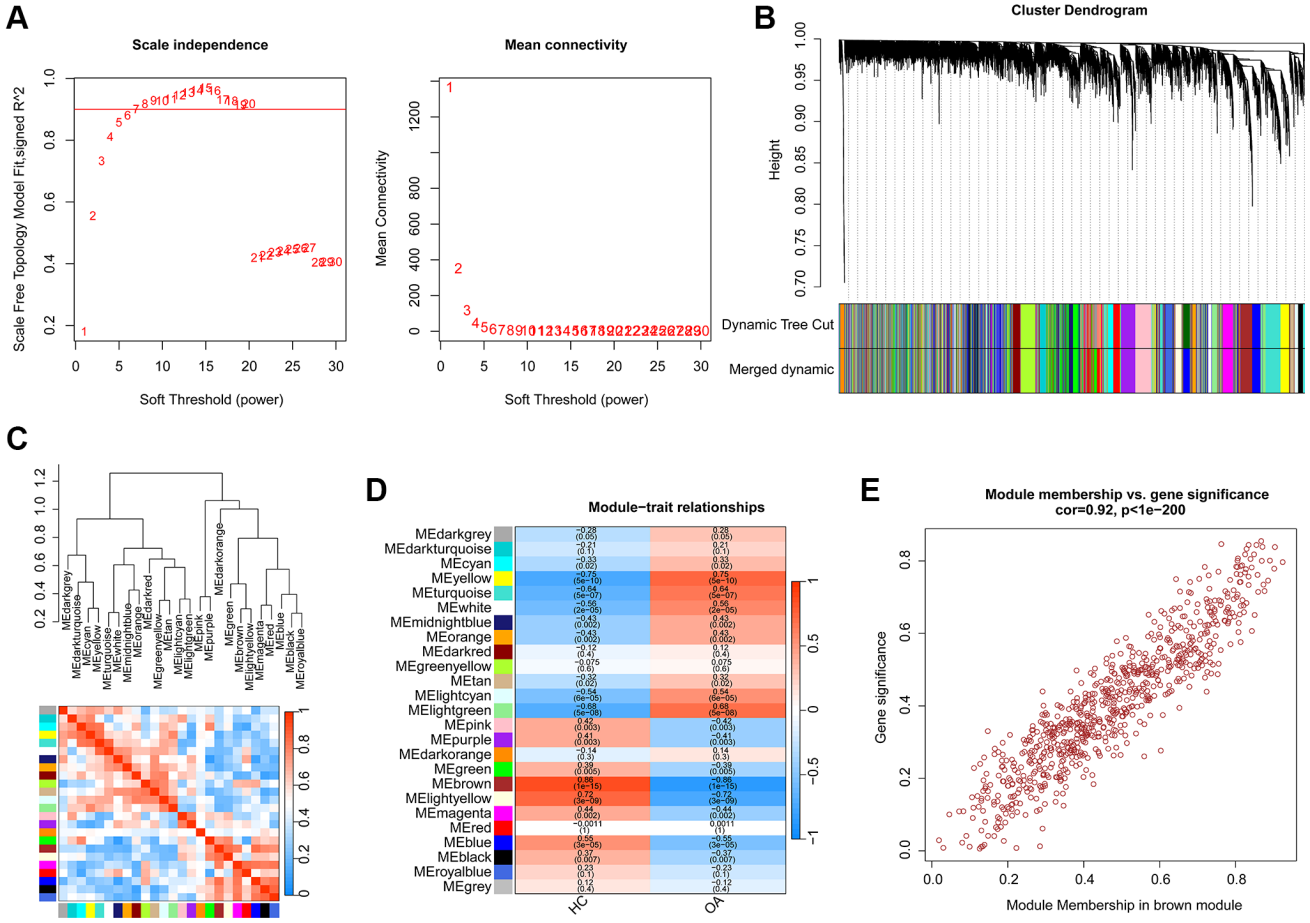
**Establishment of WGCNA model for selecting the pivotal gene module in OA.** (**A**) Scale independence and mean connectivity. (**B**) The height of different gene modules and dynamic tree cut. (**C**) Potential association of 25 unique gene modules. (**D**) Correlation analysis of 25 unique gene modules and clinical features. (**E**) The relationship of module membership and gene significance in brown module.

A total of 25 HC and 25 OA samples were enrolled to identify the pivotal gene module for OA based on the WGCNA. Firstly, the samples were clustered to exclude the abnormal samples. Based on the filter condition of scale-free topology (R^2^) set at >0.85, we constructed a WGCNA network with the soft threshold (power) selected as β = 6 (Figure 2A). With the height of gene modules set at 0.25, the feature genes were divided into the 25 inimitable gene modules (Figure 2B). Correlation analysis suggested the stable independence between the 25 inimitable gene modules (Figure 2C). Thereafter, we further evaluated the association between the clinical trait and gene modules and the result of module-trait relationships illustrated that the brown gene module was positively associated with HC but negatively associated with OA (Figure 2D). The scatter plot of brown gene module illustrated a strong correlation between the module membership and gene significance (r = 0.92, *p* < 1e-200), and was selected for the subsequent analysis (Figure 2E).

### Analysis of the pivotal DE-ERRGs and key molecular pathways

**Figure 3:**
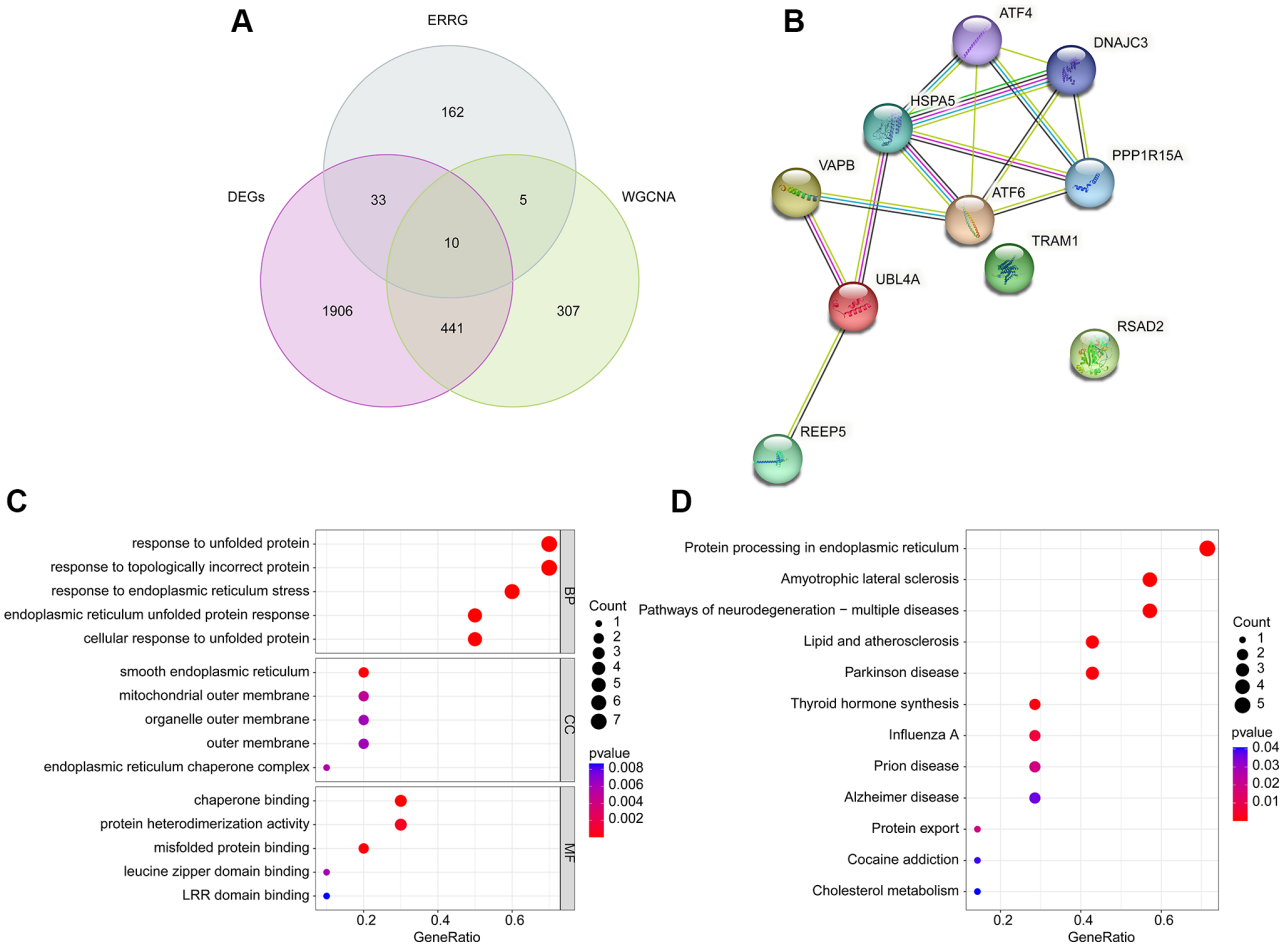
**Identification of pivotal DE-ERRGs and molecular pathway enrichment analysis.** (**A**) Selection of pivotal DE-ERRGs via differential expression analysis and WGCNA. (**B**) PPI network analysis of 10 pivotal DE-ERRGs. (**C**, **D**) Molecular function analysis of 10 pivotal DE-ERRGs.

Utilizing the differential expression analysis and WGCNA network (brown module), we observed the 10 overlapping DE-ERRGs were considered as the pivotal DE-ERRGs for OA (Figure 3A). The PPI network revealed a strong relationship of 10 pivotal DE-ERRGs (Figure 3B). To further investigate the potential molecular function of pivotal DE-ERRGs, we performed the GO ang KEGG enrichment analysis. GO enrichment results suggested that the 10 pivotal DE-ERRGs was enriched in response to unfolded protein, response to topologically incorrect protein, smooth endoplasmic reticulum, mitochondrial outer membrane and chaperone binding, while the KEGG enrichment analysis revealed that protein processing in endoplasmic reticulum, amyotrophic lateral sclerosis and pathways of neurodegeneration- multiple diseases may medicated the role of DE-ERRGs in OA (Figure 3C, 3D).

### Characteristic DE-ERRGs biomarker identification for OA

**Figure 4:**
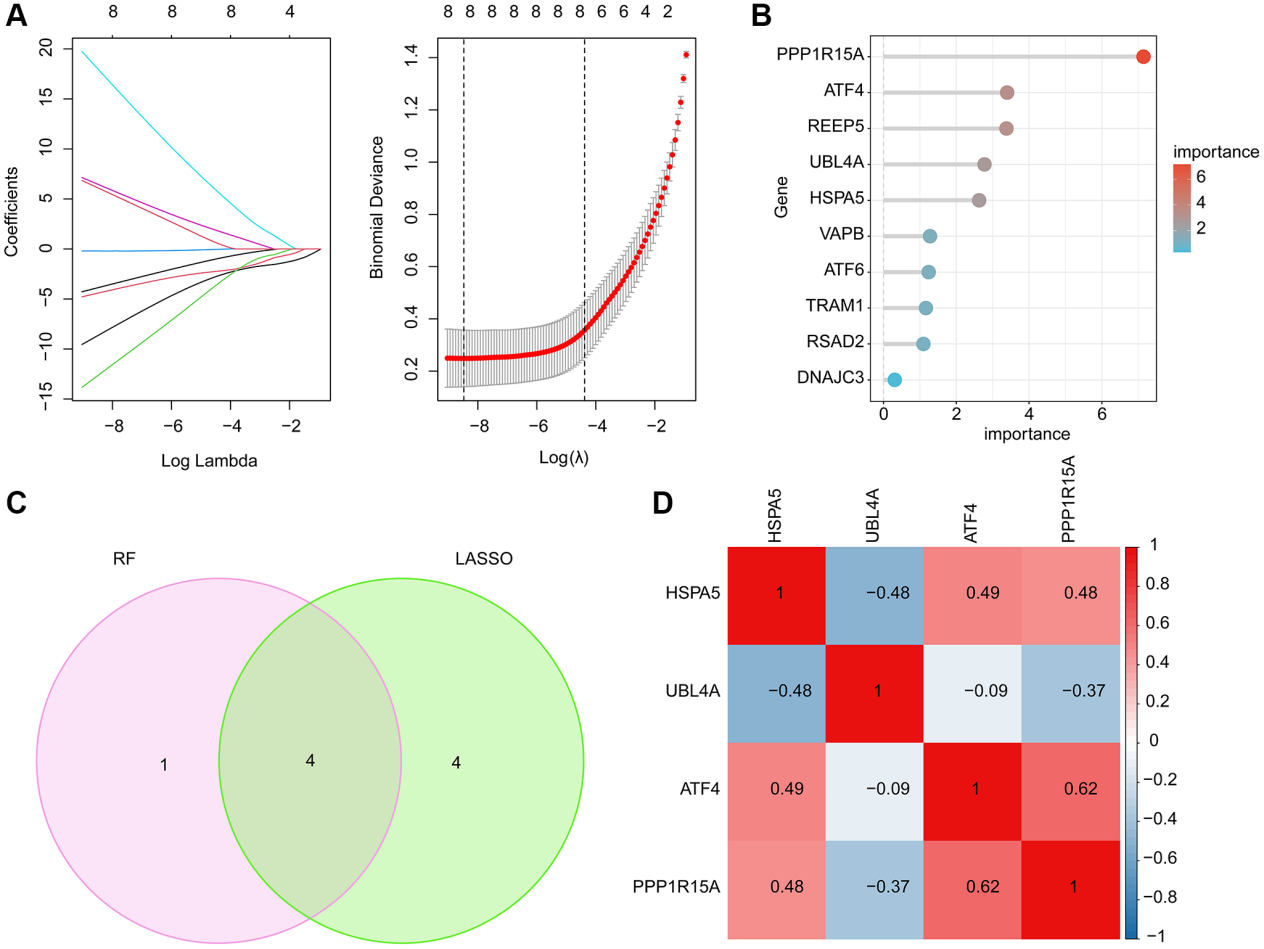
**Feature ERRGs biomarkers identification using LASSO and RF machine learning algorithm.** (**A**) LASSO algorithm for selecting feature ERRGs related biomarkers. (**B**) The importance ranking of 10 pivotal DE-ERRGs via RF algorithm. (**C**) Identification of feature ERRGs biomarkers via RF and LASSO machine learning algorithms. (**D**) Pearson correlation analysis of HSPA5, UBL4A, ATF4 and PPP1R15A.

LASSO and RF machine learning algorithms were performed to identify the characteristic ERRGs-related biomarkers for OA. Based on the expression of 10 pivotal DE-ERRGs, the LASSO algorithm revealed the coefficients of each variable and 8 feature variables was selected with the minimum log lambda (λ = 8) (Figure 4A). According to the RF algorithm, the 10 pivotal DE-ERRGs were ranked according the variable importance and 5 important variables were identified (Figure 4B). By Venn diagram, 4 overlapping DE-ERRGs were considered as the feature biomarkers for OA, involving HSPA5, UBL4A, ATF4 and PPP1R15A (Figure 4C). The Pearson correlation analysis of 4 ERGGs feature biomarkers illustrated that HSPA5 was negatively associated with UBL4A, while was positively associated with ATF4 and PPP1R15A; UBL4A was negatively correlated with ATF4 and PPP1R15A; while ATF4 was positively linked with PPP1R15A (Figure 4D).

### Model effectiveness assessment and nomogram construction of feature ERRGs biomarkers

**Figure 5:**
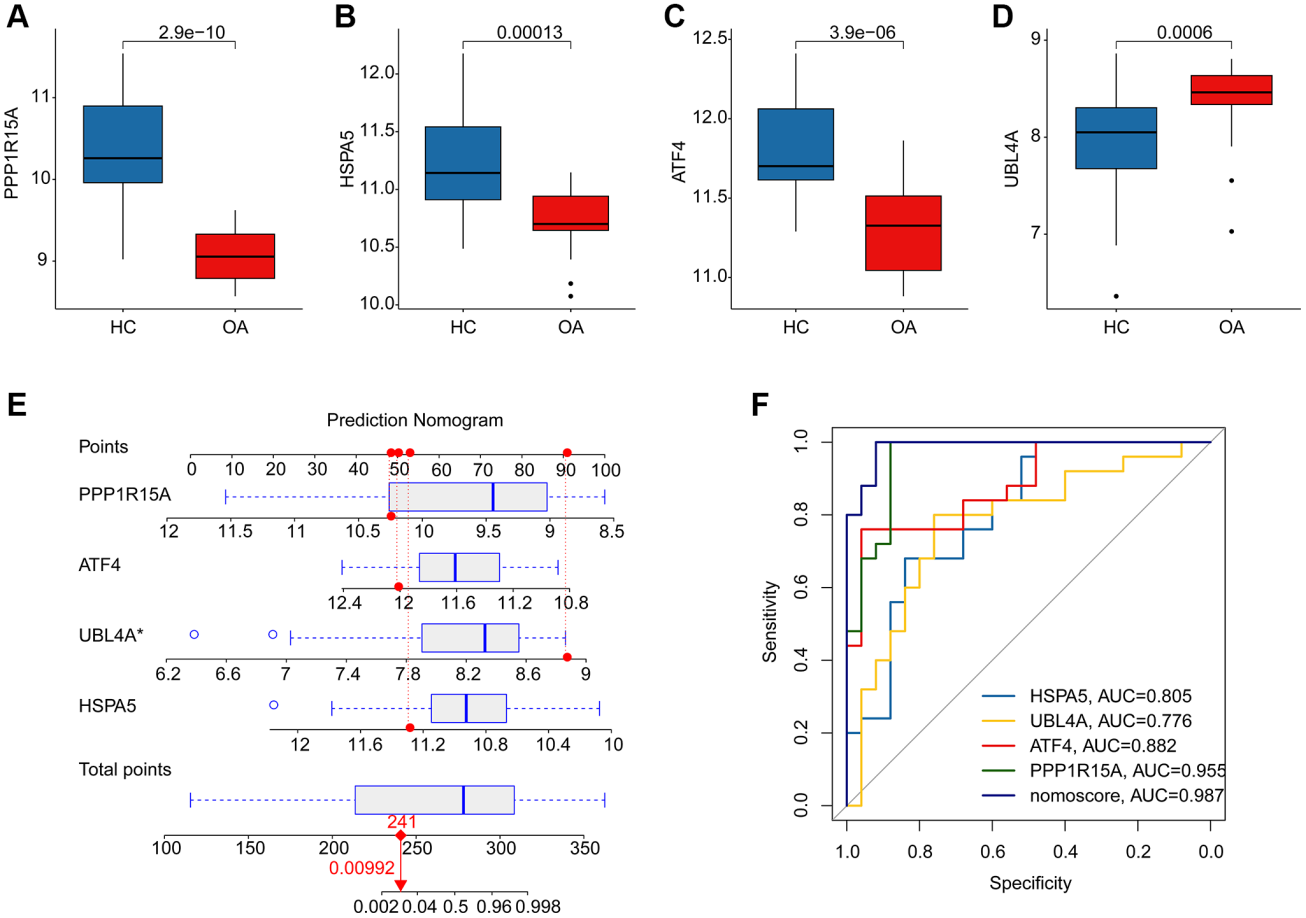
**Diagnostic effectiveness evaluation and nomogram construction based on the ERRGs related biomarkers.** (**A**–**D**) The expression profile analysis of HSPA5, UBL4A, ATF4 and PPP1R15A in HC and OA groups. (**E**) Nomogram construction based on the four ERRGs related biomarkers. (**F**) Diagnostic effectiveness evaluation of HSPA5, UBL4A, ATF4, PPP1R15A and nomogram score.

We further evaluated the diagnostic effectiveness of the ERRGs related biomarkers in OA and the expression profile results suggested that the expression of HSPA5, ATF4 and PPP1R15A in HC group was greatly overexpressed, while the expression of UBL4A was significantly overexpressed in OA group (Figure 5A–5D). According to the expression profile of the four ERRGs related biomarkers, we established a newly nomogram model to evaluate the diagnostic effectiveness for OA (Figure 5E). The ROC analysis result indicated that the AUC of HSPA5, UBL4A, ATF4 and PPP1R15A was 0.805, 0.776, 0.882 and 0.955, respectively. Notably, the AUC of nomogram model was 0.987, which was higher than HSPA5, UBL4A, ATF4 and PPP1R15A, illustrating a satisfactory diagnostic ability of nomogram model for OA (Figure 5F).

### Immune microenvironment characteristic and GSEA analysis

**Figure 6:**
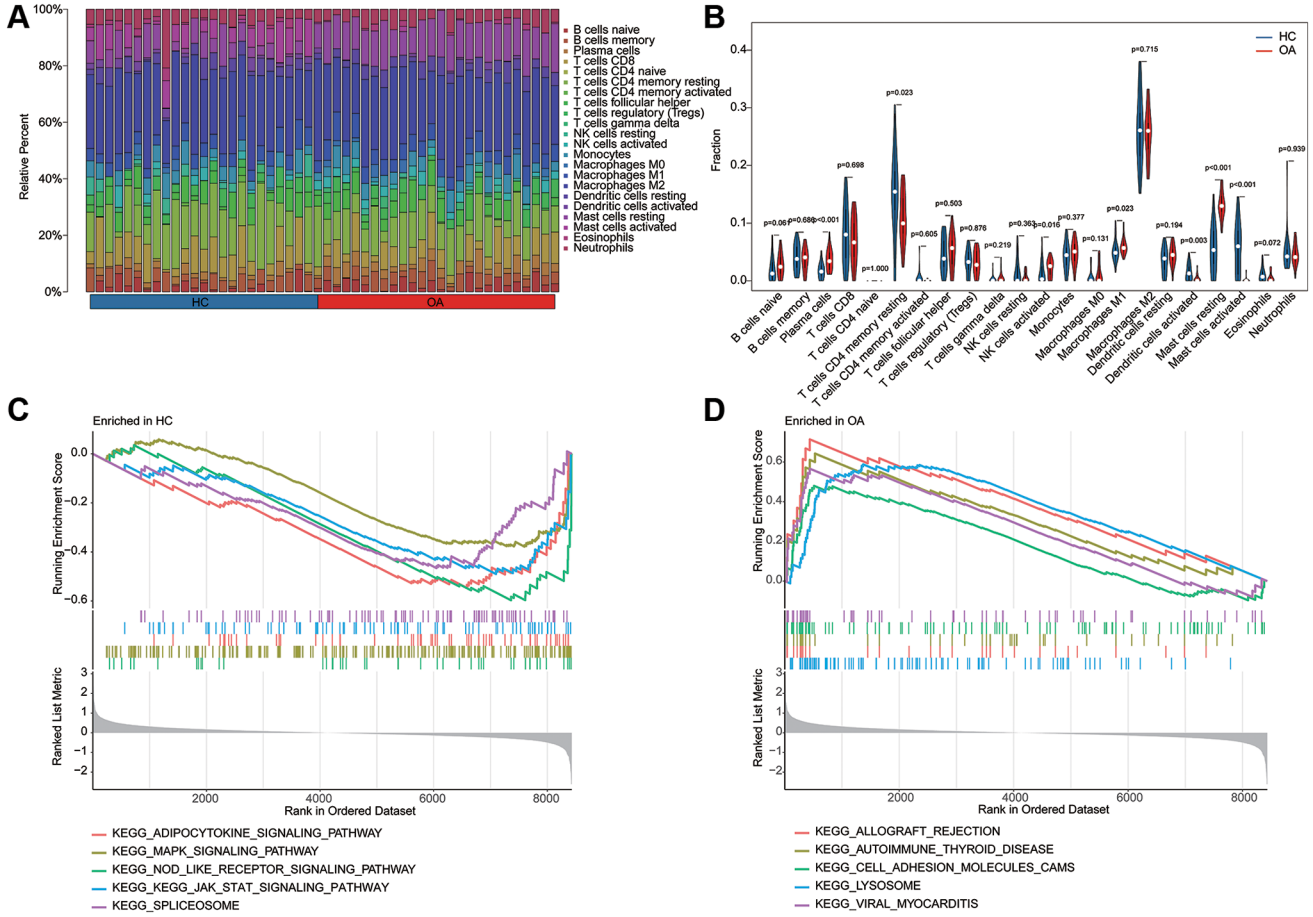
**Estimation of immune microenvironment characteristic and KEGG related GSEA analysis.** (**A**) The evaluation of 22 immune cell subtypes of HC and OA groups based on the CIBERSORT estimation algorithm. (**B**) The quantitative analysis of relative percent of 22 immune cell subtypes in HC and OA groups. (**C**, **D**) KEGG related pathway analysis in HC and OA groups based on the GSEA analysis.

Utilizing the signature of 22 immune cell subtypes, we evaluated the proportion of 22 immune cell of each HC and OA sample by CIBERSORT algorithm (Figure 6A, 6B). The quantitative results of 22 immune cells indicated that the relative percent of plasma cells, NK cells activated, macrophages M1 and mast cells resting in OA group was significantly higher, whereas the relative percent of T cells CD4 memory resting, Dendritic cells activated and mast cells activated in HC group was remarkable up-regulated than the OA group. In HC group, the GSEA result suggested that adipocytokine signaling pathway, MAPK signaling pathway and NOD like receptor signaling pathway was up-regulated; however, the lysosome, allograft rejection and cell adhesion molecules cams was up-regulated in OA group (Figure 6C, 6D).

**Figure 7:**
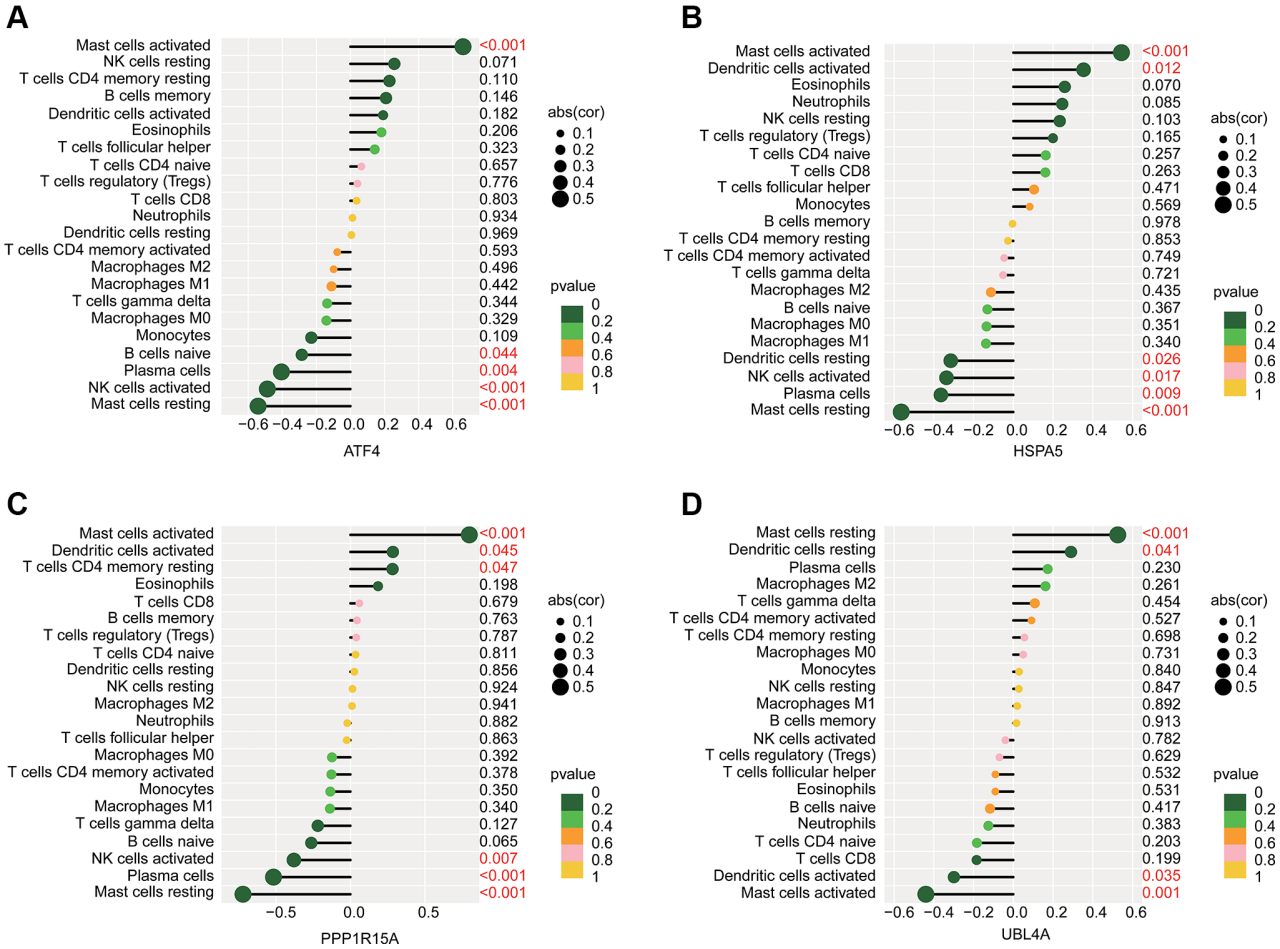
**Potential association analysis of ERRGs related biomarkers and immune microenvironment characteristic.** The lollipop plot shows the association of immune microenvironment and (**A**) ATF4, (**B**) HSPA5, (**C**) PPP1R15A and (**D**) UBL4A.

We further predicted the potential association of ERRGs related biomarkers and immune microenvironment characteristic using the Pearson correlation algorithm. As illustrated in Figure 7A–7D, we observed that ATF4 was positively associated with mast cells activated and negatively associated with mast cells resting, NK cells activated, plasma cells and B cells naïve; HSPA5 was positively correlated with dendritic cells activated and mast cells activated but negatively correlated with mast cells resting, plasma cells, NK cells activated and dendritic cells resting; PPP1R15A was positively correlated with T cells CD4 memory resting, dendritic cells activated and mast cells activated, while was negatively associated with mast cells resting, plasma cells and NK cells activated; UBL4A was positively correlated with dendritic cells resting and mast cells resting but negatively correlated with mast cells activated and dendritic cells activated.

### *In vitro* validation of ERRGs related biomarkers

**Figure 8:**
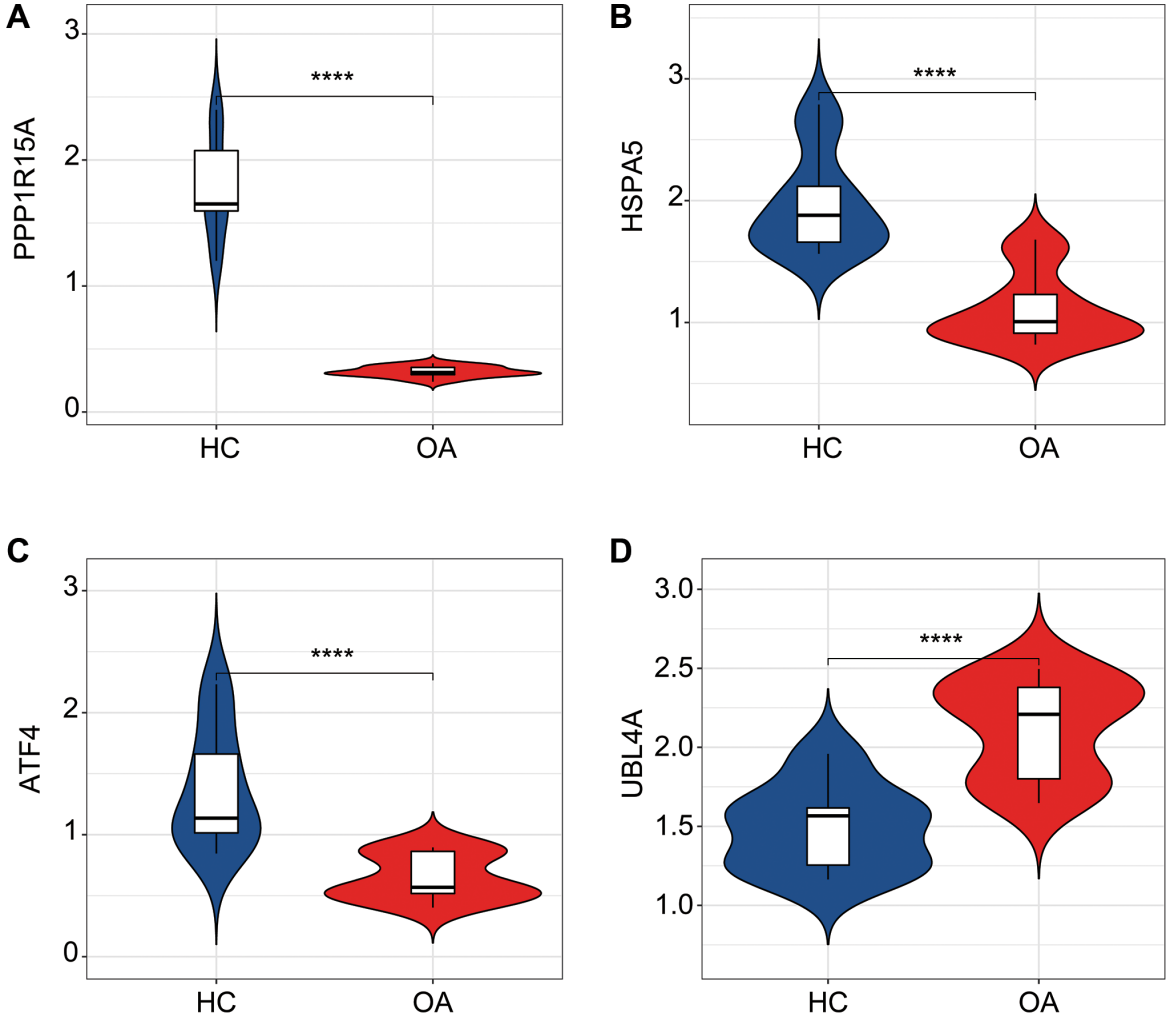
**qRT-PCR analysis of the four ERRGs related biomarkers in OA and HC groups.** The expression profiler of (**A**) ATF4, (**B**) HSPA5, (**C**) PPP1R15A and (**D**) UBL4A in HC and OA groups. ^*^p < 0.05, ^**^p < 0.01, ^***^p < 0.001, ^****^p < 0.0001.

We further validated the expression profiler of the four ERRGs related biomarkers in the HC and OA samples. The expression results revealed that the HC group had higher expression of ATF4, HSPA5 and PPP1R15A, whereas the expression of UBL4A in OA group was significantly higher than HC group (Figure 8A–8D).

## DISCUSSION

In this study, we screened four ERRGs with OA severity and progression, evaluated their effects on immune infiltration, and verified their abnormal expressions in OA patients through *in vitro* experiments.

Among the four ERRGs we screened, PPP1R15A had the most significant effect on OA. PPP1R15A is a key factor in the integrative stress response of mammals [26]. PPP1R15A mediates dephosphorylation of eIF2α [27, 28]. When eIF2α is phosphorylated, global protein synthesis is reduced, which is beneficial for cell survival and recovery [29]. In contrast, dephosphorylation of eIF2α allowed the cells to resume normal protein synthesis processes [30, 31]. Our results showed reduced PPP1R15A levels in patients with OA. PPP1R15A can direct the breakdown of the unfolded protein response (UPR) and lead to the restoration of normal ribosome activity. UPR is a direct result of endoplasmic reticulum (ER) stress [32]. Mice lacking PPP1R15A activity were healthier than wild-type controls, had improved insulin sensitivity and were more resistant to ER stress [33] and inhibited PPP1R15A to protect cells from ER stress-induced apoptosis [34]. At the same time, PPP1R15A’s promoting effect on Cd8+ T cells has also been reported [35], which is consistent with our observed immune infiltration results. Our data demonstrate the role of PPP1R15A in OA development from a new perspective.

UBL4A is essential for the mitochondrial fusion process under nutrient deprivation stress [36]. Meanwhile, as a ubiquitin ligase-associated protein, UBL4A is involved in proteasome degradation and mediates DNA damage signaling and cell death [37, 38]. UBL4A plays an important role in the development of immune dysfunction and subsequent abnormal bone metabolism. UBL4A contributes to the development of inflammatory diseases by regulating NF-CUMB signaling in macrophages and dendritic cells [39]. UBL4A knockout mice showed mild kyphosis and scoliosis with dysregulation of osteoblastogenesis and chondrogenesis [40]. UBL4A knockout mice resist collagen-induced arthritis by regulating the balance of Th1, Th17, and regulatory T cells in the T cell subpopulation [41]. We observed that the higher expression of UBL4A in OA patients may be related to the role of UBL4A in promoting inflammation and abnormal bone metabolism reported in the literature.

ATF4 is a member of the activating transcription factor (ATF)/cyclic adenosine phosphate Responsive element binding (CREB) family and plays a key role in the regulation of osteoblast function [42]. ATF4 accumulates in osteoblasts and is a specific activator of osteocalcin specific element 1 (OSE1), which is particularly active in osteoblasts [43, 44]. In addition to participating in amino acid metabolism, ATF4 plays an important role in type I collagen synthesis and transcriptional control of several major osteoblast genes [45, 46]. ATF4 is involved in various diseases related to bone metabolism, including Coffin-Lowry Syndrome [44]. In OA, AFT4 is associated with RUNt-associated transcription factor 2 (Runx2), which is essential for chondrocyte hypertrophy, and regulates osteoblast differentiation and chondro-development [47–49].

HSPA5, member A of the heat shock protein family (HSPA5), is a chaperone member mainly expressed in ER [50]. HSPA5 is involved in UPR process and promotes cell survival under ER stress [51]. Recently, HSPA5 was found to be a suppressor of the ferroptosis process [52, 53]. In OA, inhibition of HSPA5 expression can degrade GPX4, thus promoting ferroptosis in chondrocytes. However, upregulated HSPA5 expression could inhibit inflammatory damage and ferroptosis, thus alleviating OA progression [54]. Similarly, we observed that compared with the control group, the expression level of HSPA5 in OA patients was significantly reduced, which also suggested the role of HSPA5 and the ferroptosis involved in OA disease progression.

In the process of OA development, low-grade inflammatory processes formed by immune infiltration are involved. Our immunoinfiltration results showed that OA patients had significantly increased M1 macrophage infiltration levels compared with healthy controls. The number of macrophages in the synovial membrane of OA increased and was associated with OA disease progression and pain [55]. In addition, the disturbance of macrophage polarization in synovial tissue may contribute to the occurrence and progression of OA [56]. The degree of imbalance of the M1/M2 type of macrophage polarization is helpful in evaluating the severity of knee OA [57]. Further studies showed that the activated synovial macrophages in OA patients were mainly M1 macrophages, and the polarization of macrophages to M2 would weaken the development of OA, suggesting that the increase of M1 synovial macrophages may be the key reason for the deterioration of OA, which is consistent with the phenomenon we observed [58]. M1 macrophages can be induced by IFN-γ and TNF-α *in vitro* [59]. Because of its ability to produce pro-inflammatory cytokines including TNF-α and IL-1, M1 macrophages are known as pro-inflammatory macrophages [60]. The possible mechanism by which M1 macrophages affect OA is that M1 macrophages with increased content in OA synovium can secrete more cytokines. Subsequent chronic inflammation leads to cartilage degradation and osteophyte formation [61].

The results of DE-ERRGs pathway enrichment analysis showed that UPR was the most relevant pathway in biological process, suggesting the possible role of UPR in OA. UPR is induced by loading of unfolded or misfolded proteins that accumulate in ER and is intended to restore ER homeostasis by initiating apoptosis when ER stress persists [51]. UPR plays a crucial role in cartilage formation [62]. In chondrocytes and osteoblasts, bone morphogenetic protein 2 (BMP2) is an activator of UPR signaling [63]. Severe chondrodysplasia was observed in BMP2-knockout mice, accompanied by disturbances of growth plate chondrocytes [64]. In addition, the aforementioned ATF4 is also regulated by BMP2 and is involved in the expression of UPR transcription factors [63]. Targeting UPR has potential value in improving symptoms in cartilage-related diseases [65].

From targeted therapy to molecular classification and patient stratification to prognostic prediction, microarray has been shown to have important clinical applications in a variety of diseases including OA [66, 67]. In this study, we screened four ERRGs associated with OA progression through microarray analysis of a public database and verified their abnormal expression in OA patients. By analyzing the differentially expressed genes from different sources and involved pathways of OA, we can not only clarify the potential genetic mechanism of OA pathogenesis, but also explore potential OA targeted drug therapeutic targets [66, 68, 69]. Limited to the condition of public database, the selection bias of race and region existed in this study. In the future, further large-scale, multi-center analyses will better analyze the reliability and potential clinical application value of the selected targets. In addition, this study did not involve mechanism studies, and further molecular mechanism studies in the future can better elucidate the influence of different expressed genes on OA.

## Supplementary Materials

Supplementary Table 1

Supplementary Table 2
