# Supplementary material for: Machine learning-based endoplasmic reticulum-related diagnostic biomarker and immune microenvironment landscape for osteoarthritis
Source: Aging (Albany NY). 2024 Feb 28;16(5):4563–78. doi: 10.18632/aging.205611 (PMC10968715; doi:10.18632/aging.205611)
Supplement: Supplementary Table 2 [file aging-16-205611-s002.pdf]

SUPPLEMENTARY TABLE

Supplementary Table 2. The primer sequences of four ERRGs related biomarkers.

UBL4A	Forward Primer	AGATGGGAAACGACTCTCGGA
	Reverse Primer	CGCCTTCTTCTAGTAGCACCTT
PPP1R15A	Forward Primer	ATGATGGCATGTATGGTGAGC
	Reverse Primer	AACCTTGCAGTGTCCTTATCAG
HSPA5	Forward Primer	CATCACGCCGTCCTATGTCG
	Reverse Primer	CGTCAAAGACCGTGTTCTCG
ATF4	Forward Primer	ATGACCGAAATGAGCTTCCTG
	Reverse Primer	GCTGGAGAACCCATGAGGT
GAPDH	Forward Primer	TGTGGGCATCAATGGATTTGG
	Reverse Primer	ACACCATGTATTCCGGGTCAAT
